# Both immune hormone IL-10 and parathormone tend to increase in serum of old osteoporotic people independently of 1, 25 dihydroxy vitamin D3

**Published:** 2016

**Authors:** Mohamad Teimourian, Zeinab Jafaraian, Seyed-Reza Hosseini, Mahsa Rahmani, Mogjan Bagherzadeh, Azin Aghamajidi, Ali Bijani, Hajighorban Nooreddini, Amrollah Mostafazadeh

**Affiliations:** 1 Social Determinants of Health Research Center, Health Research Institute, Babol University of Medical Sciences, Babol, Iran.; 2 Immunology Department, Faculty of Medicine, Babol University of Medical Sciences, Babol, Iran.; 3 Non-Communicable Pediatric Diseases Research Center, Health Research Institute, Babol University of Medical Sciences, Babol, Iran.; 4 Department of Radiology, Faculty of Medicine, Babol University of Medical Sciences, Babol, Iran.; 5 Cellular and Molecular Biology Research Center, Health Research Institute, Babol University of Medical Sciences, Babol, Iran.

**Keywords:** Osteoporosis, IL-10, IL-17A, Parathormone, 1, 25dihydroxy vitamin D3, Elderly

## Abstract

**Background:**

In this study, we determined the serum levels of IL-17A and IL-10 in context with 1, 2 dihydroxy vitamin D3, parathormone and Ca^2+^/Pi ions to investigate their pathological or protective roles respectively in bone metabolism.

**Methods:**

The bone mineral density (BMD) was determined for 1203 participants using energy X-ray absorptiometry. Subjects with a history of diseases and using bone metabolism medications were excluded and finally serum IL-10 was measured in 82 osteoporotic and 74 healthy individuals (mean age ±SD of 71.04±6.9 and 68.58±6.9 respectively). Also, the serum level of IL-17A was assessed in 42 osteoporotic and 39 non-osteoporotic subjects (mean age±SD of 69.40±6.7 and 70.77±7.1, respectively). Serum levels of 1, 25-dihydroxyvitamin D3, Ca^2+^/Pi ions and parathormone were extracted from AHAP cohort data bank.

**Results:**

IL-17A was detectable in 7.42(16.67%) osteoporotic subjects and 3.39(7.69%) normal subjects. Surprisingly, patient subjects exhibited a higher level of serum IL-10 than normal subjects (P=0.023). We found that the serum parathormone levels tend to increase in patient group (P=0.003) in comparison to normal control with no correlation with Il-10 levels. There was no significant difference between the two groups in the serum levels of 1, 25-dihydroxyvitamin D3, Ca^2+^and P_i_ ions.

**Conclusion:**

In reaction to chronic inflammation old osteoporotic patients independent of 1, 25 dihydroxy vitamin D3 may produce a higher level of IL-10 to dampen production of inflammatory cytokines including IL-17A which in turn leads to speeding up parathormone production ultimately reaching a new homeostasis status in bone metabolism with normal serum Ca^2+^ /Pi ions.

**Bone as one of the hardest tissues in the human body, can be transformed into a brittle structure through enzymatic digestion by osteoclast, a bone marrow-derived macrophage, during a process so-called osteoporosis. Bone synthesis (ossification) is a result of a tightly regulated interplay between bone forming cell (osteoblasts) and bone resorbing cell (osteoclasts) in which the latter is produced via a process named osteoclastogenesis. This interplay occurs via a direct osteoblast and osteoclast contact (** 1 **) as well as through the secretion of some hormone-like substances called cytokine which are mainly produced by immune cells. Receptor activator of NF-κB ligand (RANKL) and macrophage-colony stimulating factor (M-CSF) which are synthesized by osteoblasts and immune cells are the two main players in this crosstalk (** 2 **,** 3 **,** 4 **).**

Contrary to these cytokines, osteoprotegerin (OPG) as a decoy receptor and bone protector cytokine receptor binds with RANKL to inhibit receptor activator of NF-κB (RANK) activation which in turn results in the inhibition of osteoclastogenesis and bone resorption (2, 3). One important point that should be noticed here is that this crosstalk is a dynamic process in whole lifespan of human being. Moreover, it is an age and sex-dependent phenomenon. We well know that from early embryonic life (third month) to late adolescence (18-20 years old), the activity of osteoblast prevailed to osteoclastogenesis and after that time, the levels of bone formation and bone destruction reach to an equilibrium level which persists for almost a decade. Around the fourth decade of human life, this equilibrium shifts to outpace of osteoclastogenesis (bone destruction) over bone formation that in sequence results in a pre-osteoporosis status called osteopenia which ultimately progress to osteoporosis (4). 

In an adult female, bone resorption will accelerate more than male due to estrogen deficiency which occurs naturally in postmenopausal women (4). 

In addition, bone metabolism is greatly influenced by some environmental factors like sunlight, infections as well as some nutritional elements like vitamin D, phosphorous (Pi) / calcium (Ca^2+^) ions. Indeed many of calcium metabolism regulating hormones and cytokines, such as 1, 25 dihydroxy vitamin D3, parathormone( PTH), prostaglandin E2 (PGE2) and interleukin-11(IL-11) play a dual role in inhibiting OPG synthesis and increasing RANKL production (5, 6). 

In contrast to these factors estrogen appears to downregulate the production of RANKL and consequently the RANKL-mediated osteoclastogenesis (5). Regardless, apart from this natural occurrence of negative bone turn over, osteoporosis can be observed in some inflammatory diseases such as inflammatory bowel disease (IBD) (7) and rheumatoid arthritis (8). Additionally, RANKL directly regulates the pro-inflammatory cytokines production in macrophages and consequently regulates the inflammatory and immune responses (9). 

Indeed osteoporosis can be considered as an inflammatory disease. Thus, pro and anti-inflammatory cytokines appear to have pro and anti-osteoporotic activities, respectively. Accordingly, interleukin-17A is one of the most important proinflammatory cytokines which is produced by Th17 cells (10, 11). Although the involvement of interleukin -17 (IL-17) in pathogenesis of osteoporosis has already been shown in different research levels such as ovariectomized mouse model, bone cells and genetic research, human-related studies about this subject are still few (12, 13). 

On the other hand, as a pleiotropic immunomodulatory cytokine, interleukin-10(IL-10) can be considered as an osteoprotective protein .This cytokine also downregulates the IL-17 expression (14-16). Interestingly, the anti-inflammatory and anti-aging protein, so-called alpha-klotho which is expressed by T-lymphocyte (17) and kidney (18) can upregulate the IL-10 expression in experimental model (19). 

Alpha-klotho protein can accelerate calcium absorption from kidney distal convoluted tubules and also is able to induce the parathormone secretion and through these mechanisms it plays an important role in Ca^2+^/P_i_ homeostasis (18). Together it means that IL-10 may have an indirect role in Ca^2+^/P_i_ homeostasis. In addition, there is some convincing evidence implicating an immunomodulatory role for another calciotropic agent i.e. 1, 25-dihydroxyvitamin D3 in different autoinflammatory diseases like experimental autoimmune encephalomyelitis, multiple sclerosis and rheumatoid arthritis via IL-10-IL-10 receptor signaling (20-23). Hence, determining the correlation between serum IL-10/IL-17 and vitamin D levels can be a useful achievement in understanding immunological aspects of osteoporosis as well as for its management especially in elderly people with vitamin D supplementation. 

In the present study, we investigated the correlation between serum IL-17/ IL-10 concentrations and osteoporosis in context with1, 25-dihydroxyvitamin D3, Ca^2+^/P_i_ ions and parathormone in old people who have already participated in the cohort project named Amirkola Health and Ageing Project(AHAP) cohort.

## Methods

The osteoporotic and normal control subjects of this study were selected from Amirkola Health and Ageing Project (AHAP) which was done from April 2011 to July 2012 in elderly people aged 60 years and above, residents of Amirkola city located in northern Iran near the Caspian Sea (24).

In total, 1616 older people participated in this project and the bone mineral density (BMD) of 1203 subjects has been measured by dual energy x-ray absorptiometry (DEXA scan) with Lexxos bone densitometer (Lexxos France) in femur neck and lumbar spine (L2-L4). According to the conventional World Health Organization (WHO) definition, the subjects with T-score≤-2.5 and T-score≥-1 were considered as the osteoporotic and normal individuals respectively (25). 

Individuals with a history of diseases and using bone metabolism medications such as hyperthyroidism, hyperparathyroidism, diabetes mellitus, liver disease, renal disease, corticosteroids, anticonvulsants, heparin sodium were excluded from the study and finally, of the 585 subjects (266 females aged 60-89 and 319 males aged 60-89), 154 subjects were included in the present study as patient and control groups. Serum sample has been already prepared from these subjects and stored at -80 c until the day of experiment.

IL-10 was determined by sandwich enzyme linked immunosorbent assay (ELISA)* (Diacolone*, France and R &D, USA) in serum of 82 osteoporotic (45 females aged 60-84 and 37 male aged 60-74) and 72 non-osteoporotic control subjects (26 female age range 60-84 and 46 male age range 60-86). 

Besides, using Elisa kit (*Diacolone*, France) the serum level of IL-17A was measured in 42 osteoporotic (22 Females aged 60-80 and 20 males aged 60-84) and 39 non-osteoporotic people (19 females aged 60-80 and 20 males aged 62-86).

Serum levels of 1, 25-dihydroxyvitamin D3, Ca^2+^/P_i_ ions, parathormone which have already been determined by routine biochemistry clinical laboratory methods were extracted from AHAP cohort data bank. We used the Kolmogorov-Smirnov test for normality of variables. Mann-Whitney U-test or t- test to compare the means of variables. Bivariate Pearson correlation was determined using SPSS software Version 21. 

## Results

**Figure 1:**
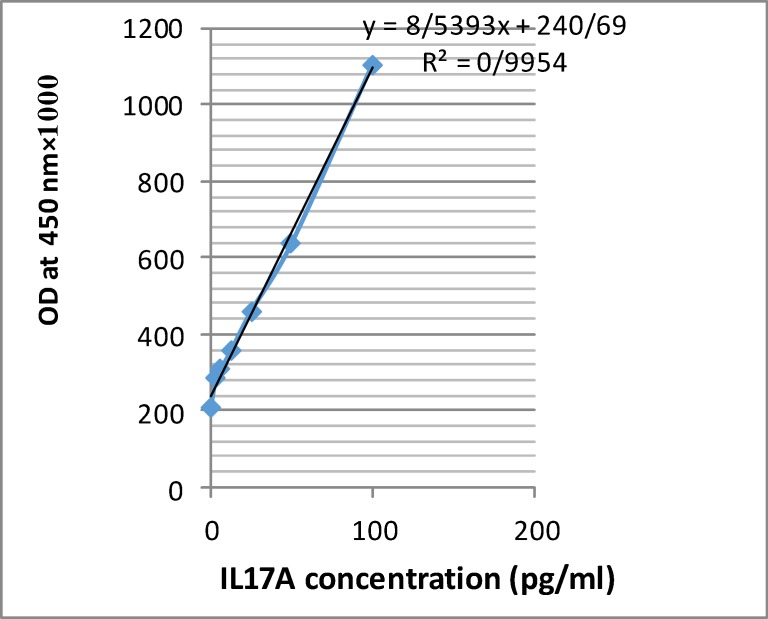
IL17 Elisa standard curve

To investigate the possible pathogenic role of IL-17A in osteoporosis, we determined the serum levels of this cytokine in osteoporotic and normal control groups. IL-17A was detectable only in 10 out of 81 (12.35%) of total serum specimens (prepared from 7 out of 42 (16.67%) in osteoporotic subjects versus 3 out of 39 (7.69%) in normal subjects. We obtained a proper standard curve with a good linearity according to kit instructions (fig 1). The low detection limit value of the kit was 2.3 pg/mL.

To draw this curve, we used 100 pg/mL of recombinant IL-17A which was supplied by the ELISA kit (Diacolone, France) as the top standard concentration and then it was serially diluted two fold in phosphate buffered saline (PBS) to obtain the concentration of 50, 25, 12.5, 6.25, 3.125, 0 pg/mL and dispensed in anti-human IL-17A coated ELISA palate as duplicated wells. After adding biotinylated anti-human IL-17A antibody and washing the streptavidine horse-radish peroxidase conjugate (HRP) enzyme were dispensed into the wells. After washing and adding substrate (H2o2+teteramethyl benzidine), theoptical density (OD) values were measured at 450 nm in an ELISA reader. The correlation between IL-17A concentrations and OD was obtained maximally (r=0.995).

To investigate the possible bone protective role of IL-10, we determined the serum level of this cytokine and compare it between the patient group and control group. As it can be seen in table 1, patient group surprisingly exhibited a higher level of serum IL-10 than normal group (P=0.023).

To evaluate the bone metabolism in two groups, we compared the concentrations of two most important ions and two calciotropic hormones i.e. ca^2+^and P_i_, 1, 25-dihydroxyvitamin D3 and parathormone, respectively in our study groups, likewise, we investigated the correlation between parameters and serum IL-10 levels in two groups. We found that serum parathormone levels tend to increase in patient group (P=0.064) in comparison to normal control group (table 1). 

**Table 1:** The demographic data, mean values, standard deviation of IL10, Ca^2+^, Pi, Vit-D and PTH in studied groups

**Variables**	**Mean±SD**	**P-value**
**Age(year)**		
Normal Osteoporosis	68.58±6.979 71.04±6.964	0.910
**Interleukin-10(pg/mL)**		
Normal Osteoporosis	0.584366±0.5711010 1.484458±3.3648311	0.026
**Vit-D(I.U/mL)**		
Normal Osteoporosis	35.5246±32.24286 29.3193±24.13768	0.314
**Ca(mg/dL)**		
Normal Osteoporosis	9.3229±0.445 9.1928±0.44987	0.933
**Pi(mg/dL)**		
Normal Osteoporosis	3.8643±0.46843 3.8916±0.55463	0.142
**PTH(pg/mL)**		
Normal Osteoporosis	47.592±25.0266 56.301±37.8898	0.064*

* It reached to 0.003 when the sample size of osteoporotic and control subjects increased to whole numbers and when the osteopenic subjects (-2.5<T score<-1) were also included.

**Figure 2:**
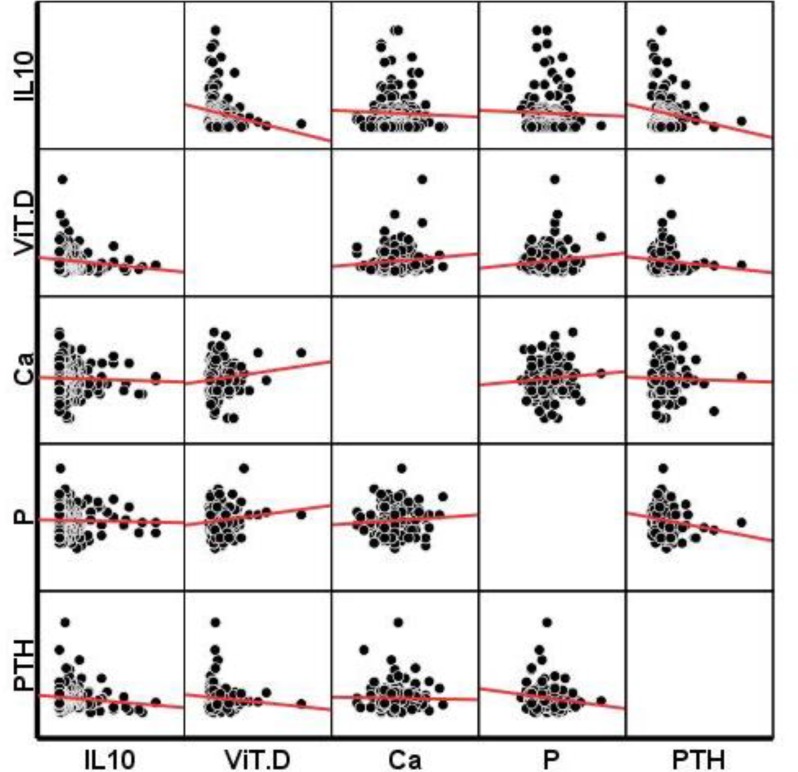
Investigation of correlation between serum IL10 ,VitD , Ca^2+^, Pi and PTH

Engrossing this difference became more obvious (P=0.003) when we increased our sample size using the whole osteoporotic and non-osteoporotic subject’s parathormone data extracted from AHAP data bank. (osteoporetic and osteopenic subjects; n=701 female/ male age range 60-89 and non-osteoporetic subject; n=332 female/ male age range 60-89). We were not able to find any statistically significant correlation between serum parathormone and IL-10 levels when we did Bivariate Pearson correlation test (figure 2). We also did not find any significant difference between the two groups in terms of serum levels of 1, 25-dihydroxyvitamin D3, ca^2+^and P_i_ ions and there was no significant correlation between these parameters involved in bone homeostasis and serum IL-10 levels (figure 2).

## Discussion

In the current study, we aimed to investigate the possible pathogenic role of IL-17A and IL-10 bone-protecting role in osteoporosis. Despite our initial assumption, we found that osteoporotic subjects exhibited a higher serum IL-10 concentration than the normal control. This finding is contrary to Gür A et al.’s study who reported the diminished IL-10 in serum of postmenopausal osteoporotic women (26). However, in the existing study, postmenopausal osteoporotic women were only about 55% of entire old osteoporotic subjects who participated in our IL-10 study. Furthermore, this finding is obviously opposite to a large number of animal studies which indicated the double role for IL-10, anti-osteoclastogenesis and pro- osteoblastogenesis in bone protection if we assumed a pathogenic role for this cytokine in osteoporosis (27-29). To the best of our knowledge, there is no report in the literature indicating an elevated level of IL-10 in osteoporotic old people. To interpret this new finding, we supposed that in compensation to chronic inflammation existed in osteoporosis subjects IL-10 synthesizing increased. 

It seems to be logical, that since 55% of serum which we used for IL-10 study prepared from postmenopausal women with mean age 68.5 years and 45 % of studied sera belonged to men with almost the same age. If we assume that the women who participated in our study reached menopausal status when they were 45-50 years old, their postmenopausal duration was between 15-20 years when the serum specimen was taken from them. We know that the maximum change in bone metabolism occurs 5-10 years after menopause (4), possibly after this time through an unknown mechanism probably via alternative RANKL-NFKB signaling pathways IL-10 expression has increased. In male subjects, the course of bone destruction is almost the same, albeit with less intensity due to a progressive decline in sex steroid hormone levels (4). 

To evaluate this hypothesis, we need further studies with a special design to cover a proper window period between premenopausal and postmenopausal period in women and as a control group almost the same period of time in men. There is some evidence in consistent with this hypothesis. Percegoni N et al. reported that serum IL-10 increasing rate in ovarietomized rats is slower than the inflammatory cytokine IL-1β (30). 

Lawrence T explained the RANKL ability to activate the nuclear factor-kappa B through alternative pathway leading to inhibition of inflammatory response by modulating the expression of anti-inflammatory cytokines such as IL-10 (31). We also found that the levels of one of the most important hormone in bone metabolism namely parathormone increased in osteoporotic patients in comparison to normal control, whereas the Ca^2+^ and P_i_ levels were almost the same in two groups. Cerdà D et al.’s study confirms this finding. They also found an elevated level (>65pg/ml) of parathormone in postmenopausal osteoporotic women with no significant difference in Ca^2+^ and1, 25-dihydroxyvitamin D3 concentrations (32). 

In a population-based study by Cheng SP et al. showed an age dependent increase in serum parathormone levels in adult (33). In spite of that, in the ongoing study the osteoporotic subjects exhibited a lower level of another important factor in bone turnover namely, 1, 25-dihydroxyvitamin D3 in comparison to normal group, yet, this difference was not statistically significant. PTH stimulates the osteoblast and liver cell to produce the inflammatory cytokine IL-6 which one after the other plays an important role in bone resorption by activating osteoclastogenesis (34). 

Besides this role, this cytokine is one of the most important cytokines in differentiation of naïve Th to Th17 cells (35). We did not investigate the inflammatory marker such as C - reactive protein (CRP) as well as serum IL-6, but our unpublished data showed the osteoporotic subjects had a slightly higher neutrophil: lymphocyte ratio wherein this ratio is considered as one of the inflammatory markers. It implicates a higher inflammatory status in these subjects. On top of that, in an attempt to investigate the pathogenic role of IL-17A in osteoporosis, although more than 87 % of subjects did not have a remarkable levels of IL-17A in their blood (the low detection limit of the kit was less than 2.3 pg/ml) the number of osteoporotic subjects with detectable level of serum IL-17A was greater than the normal control (7.42(16.67%) versus 3.39(7.69%)). To interpret this undetectable level of this cytokine in majority of cases with osteoporosis, we assumed that we did the sampling from these subjects when the production of this cytokine was downregulated due to passing of a long time from peak of osteoporotic event at least in women. For the definite verification about the IL-17A result, we need high sensitive ELISA kit (less than 1-2 pg/mL) to determine the serum level of IL-17A. We did not find any correlation between serum IL-10 levels and neither parathormone nor vitamin D3 and Ca2+/P_i_. This finding is not able to confirm the immunomodulatory role of vitamin D3 via upregulating IL-10 production. 

To interpret the observed higher levels of serum IL-10 in osteoporotic old people, we have to determine the T regulatory (T-reg) activity in these subjects. This cell is one of the most important cells in IL-10 production. There is some emerging evidence indicating that some types of T-reg cell specifically naturally occurring Tregs increase in elderly (36). 

Alternatively, klotho (anti-aging hormone) is a protein which is produced by both CD4+ T-cell and kidney tubules that can stimulate both IL-10 and parathormone production. We suggest determining the correlation between IL-10 and klotho in osteoporotic old patients in other studies. 

Taken together, the data generated by this study indicated that in response to chronic inflammation which exists in old osteoporotic individuals, the production of IL-10 as a major anti-inflammatory cytokine is independent of vitamin D3 that increased by slowing down bone destruction process resulting in a new bone homeostasis in old people. Additionally, serum parathormone level increased independently of IL-10, vitamin D, and Ca2+/P_i_ levels in these subjects. Although the percentage of IL-17A positive individuals was higher in osteoporotic old people the serum levels of this cytokine was not remarkable among the majority of old people. 

## Notes

**Funding:** The financial support of this research project was granted by the Vice-Chancellery for Research and Technology of Babol University of Medical Sciences.
